# Esophageal Stenting in the Setting of Malignancy

**DOI:** 10.5402/2011/719575

**Published:** 2011-08-08

**Authors:** Juan Carlos Martinez, Matthew M. Puc, Roderick M. Quiros

**Affiliations:** ^1^St. Luke's Hospital and Health Network, Bethlehem, PA 18015, USA; ^2^Surgical Oncology, Cancer Care Associates, St. Luke's Hospital and Health Network, 801 Ostrum Street, Bethlehem, PA 18015, USA

## Abstract

Esophageal cancer is often diagnosed at an advanced stage, with many patients found to have locoregional or metastatic disease at time of diagnosis. Because of this, cure may be unlikely, leading treatment efforts to focus more on symptom palliation and improving patient quality of life. The majority of patients with advanced disease suffer from some degree of dysphagia. Palliative efforts are therefore directed at relieving dysphagia, allowing patients to manage their oropharyngeal secretions, reduce aspiration risk, and maintain caloric intake orally. A variety of endoscopic treatment modalities have been utilized with these objectives in mind, with options determined by the location and size of the tumor, as well as the patient's expected prognosis. In this article, we review the use of endoscopically-placed stents for palliation in patients with advanced esophageal cancer. We discuss the history of stent use in such cases, as well as more recent developments in stent technology. We give an overview of some of the more commonly used stents in practice, discuss the technique of insertion, and survey the short- and long-term outcomes of stent placement.

## 1. Introduction

Esophageal cancer is a relatively rare form of cancer with an estimated 482,300 new cases in 2008 worldwide. The incidence rates of esophageal cancer vary greatly based on region, with the highest rates found in Asia, including China and Central Asia, and in Africa [1]. The USA is a relatively low-incidence area for carcinoma of esophagus, with approximately 16,640 new cases and 14,500 deaths in 2010 [2]. Most carcinomas of esophagus are diagnosed at an advanced stage, with very few patients eligible for potentially curative resection at the time of presentation, leaving palliation as a more realistic option for these patients [3]. Dysphagia is the predominant symptom in more than 70% of patients with esophageal cancer resulting in weight loss and malnutrition [4]. Other symptoms include aspiration of saliva (due to complete dysphagia) or food (secondary to esophagorespiratory fistulae), and thoracic pain caused by the invasion of an unresectable tumor. These symptoms have led to the development of a variety of treatment modalities to help maintain caloric intake and improve the quality of life remaining for patients with advanced disease. Presently, a number of endoscopy-based modalities are utilized to palliate these symptoms and will be covered in this paper. 

## 2. Background

**Figure 1:**
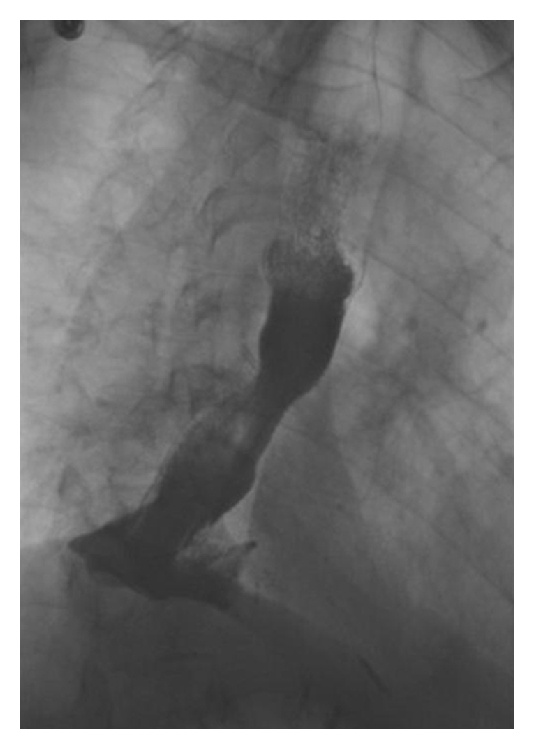
Barium swallow after stent deployment for a midesophageal stricture due to malignancy.

**Figure 2:**
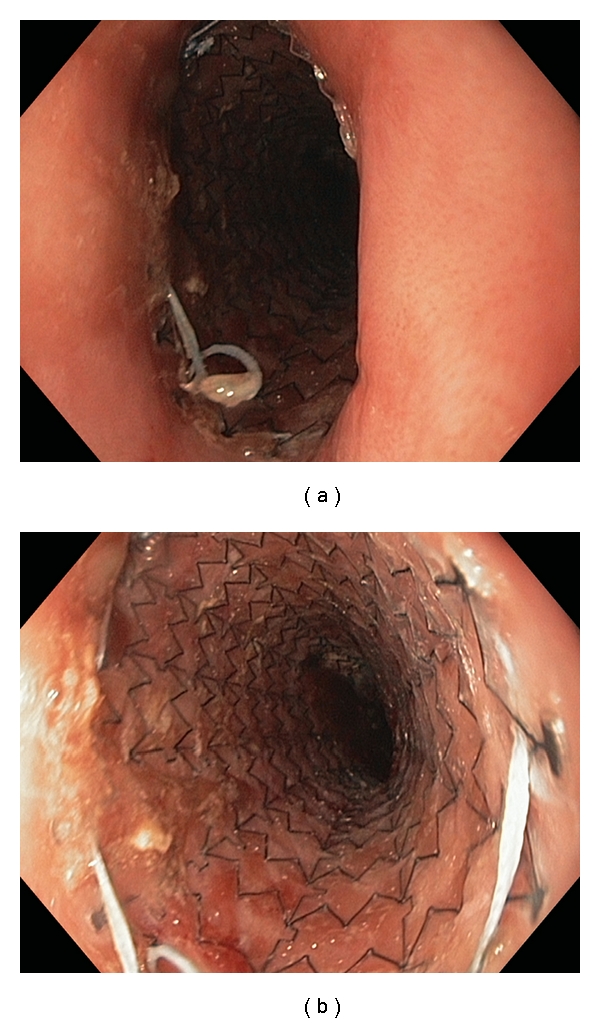
Endoscopic appearance after placement of Alimaxx stent placement. (a) shows proximal extent of stent. (b) shows reveals stent architecture.

The primary goal of esophageal stent insertion in patients with advanced disease is to relieve dysphagia and to prevent malnutrition. Compared to parenteral nutrition, endoscopic stent placement significantly improves a patient's quality of life by restoring the ability to take in food and fluids orally. Specific indications include intraluminal obstruction due to tumor, extrinsic esophageal compression due to primary or secondary tumors, refractory or recurrent esophageal strictures, malignant esophageal perforation, tracheoesophageal fistula, gastroesophageal anastomotic leaks, and tumor recurrence after surgery or chemoradiotherapy (Table 1, Figures 1 and Figures 2) [5–7]. 

Historically, esophageal intubation for obstruction due to tumor was achieved with the use of rigid polyvinyl plastic stents, either per oral pulsion or using an open traction technique, which required a laparotomy and gastrostomy. Although effective in more than 80% of patients, plastic stents were associated with complications such as migration, food impaction and perforation in up to 10% of patients [8]. Since the introduction of uncovered self-expanding metal stents (SEMSs) in the early 1990s, plastic stents have rarely been utilized. SEMS placement was found to provide immediate palliation of dysphagia in greater than 85% of patients when evaluated with a standard dysphagia scoring system [9–11]. According to a meta-analysis by Yakoub, compared to plastic stents, metal stents are associated with significantly reduced stent-related mortality (1.7% versus 11.1%), reduced esophageal perforation (1.4% versus 9.4%), and stent migration. On the other hand, tumor ingrowth through the open mesh architecture may occur in 13% of SEMS cases compared to 1.6% for when plastic stents are used [12, 13]. In response to this, the next generation of partially covered self-expanding metal stents, which added a thin silicone or plastic covering to the body of the stent to prevent tumor ingrowth was developed. Soon after it was observed that hypertrophic granulation at the uncovered ends of the stent prevented repositioning or removal of stents, making them practical only for palliation of malignant dysphagia, as stents could not be adjusted [14], this led to the introduction of fully covered self-expanding plastic stents (SEPS) in 2001. These initial SEPS exerted more radial force than their metal counterparts, causing patients some degree of discomfort. More recently, newer fully covered SEMS have been developed as an alternative due to this shortcoming.

## 3. Stent Selection

There are several commercially available expandable metal stents in the United States. Esophageal SEMS can be classified based on (1) covering (partially versus fully covered), (2) alloy material (nitinol (alloy of 55% nickel and 45% titanium) versus surgical steel versus plastic polyvinyl), (3) function (fully patent versus antireflux), and (4) biodegradability. Once malignant dysphagia has been diagnosed, the stricture must first be characterized; stent selection is then tailored to the patient based on the tumor's characteristics. SEMS come in different diameters, lengths, and deployment mechanisms that need to be considered based on several variables related to the tumor. 

### 3.1. Tumor Length

Tumor margins must be determined either endoscopically or with an esophagram. The stent must be long enough to bridge the stricture and the propensity for tumor growth around the ends must be considered. Specifically, the stent should extend 2–4 cm beyond the ends of the stricture to minimize the chances of tumor overgrowth at the ends of the stent.

### 3.2. Tumor Bulk

The luminal diameter of the obstructing tumor must allow for passage of the stent-introducer catheter. In some cases, dilatation is required to allow stent passage. The small applicator size of the self-expanding metal stents requires less aggressive dilation. 

### 3.3. Tumor Location

Midesophageal lesions are best suited for stenting. Nevertheless, stents can be also placed in the distal esophagus (albeit with increased risk of gastric reflux, regurgitation, and aspiration), and in the proximal esophagus (though the tumor must be more than 2 cm from the upper esophageal sphincter). In a small series of patients, a newly designed SEMS with a short upper flange (0.7 cm in length) was used in the treatment of proximal/cervical esophageal cancers. Stent placement was successful in these patients with no serious complications [15]. Another area of debate is the routine use of SEMS with an antireflux valve in the management of malignant dysphagia due to distal esophageal and gastric cardia malignancy; presently, the use of stents with an antireflux valve remains has yielded inconsistent results regarding efficacy of reflux prevention among recent studies [7].

### 3.4. Configuration of the Obstructive Stricture

The tortuosity of malignant strictures can pose a challenge for stent placement, since stents tend to fit best in strictures that allow the stent to remain relatively straight. Tortuosity was more problematic with early plastic stents due to their poor flexibility. Modern stents are more flexible, allowing retention of luminal patency in very tortuous strictures, with less local trauma. 

### 3.5. The Presence of Tracheoesophageal Fistulas

Covered stents can be effective at occluding the fistula tracts caused by locally invasive malignancy, facilitating closure [3].

## 4. Stent Devices

(i) SEMS are available as uncovered, partially covered, or fully covered (Table 2). There is clinical evidence to suggest that partially covered SEMS are superior to uncovered SEMS, in the palliation of unresectable obstructive esophageal cancer, mainly because tumor ingrowth through uncovered stents results in recurrent dysphagia. Vakil and colleagues reported on 62 patients in a prospective, multicentric, randomized, controlled trial. Thirty patients with malignant inoperable esophageal obstruction at the gastroesophageal junction were treated with uncovered stents, while 32 patients with comparable disease were treated with covered stents. One week after stenting the dysphagia scores improved similarly in both treatment arms. However, tumor ingrowth was significantly more likely in the uncovered stent group (9/30) than in the covered group (1/32) (*P* = 0.005). Reinterventions for tumor ingrowth were also significantly greater in the uncovered stent group (27%), as compared with 0% in the covered group (*P* = 0.002) [16]. Similarly, in another study 2 different types of SEMS (uncovered nitinol Strecker and covered ultraflex stents) were compared in the palliative treatment of 152 patients with inoperable malignant stenosis of the esophagus and cardia. Overall 88% of patients with covered stents and 54% with uncovered stents were free of symptoms during followup (*P* < 0.0001). Restenosis causing recurrent dysphagia were significantly higher in patients with uncovered stents compared to covered stents (37% versus 8%, *P* < 0.0001) despite lower rates of stent migration (0 versus 10%, *P* = 0.03) [17].

(ii) Self-expanding plastic stents (SEPSs) have been widely used for the palliation of esophageal cancer over the last decade, since they combine the advantages of SEMS with those of previously used plastic stents. In a prospective cohort study of 33 patients with malignant esophageal stenos treated palliatively with the SEPS, improvement of dysphagia was noticed in all cases. Stent occlusion occurred due to tumor overgrowth in 12.1%, stent migration was observed in 6.0%, and the reintervention rate was 21.1% [18]. Another group of investigators reported their experience on 60 patients with esophageal obstruction due to esophageal cancer (*n* = 52), lung cancer (*n* = 7), and thyroid tumor (*n* = 1), treated with the Polyflex stent. Early minor complications (e.g., chest pain, fever, gastroesophageal reflux symptoms, and incomplete usage of stent) occurred in 32% of patients, while major complications (including 3 deaths, one occurring due to pulmonary embolism and two after massive hemorrhage) were seen in 22%. Delayed stent migration occurred in 5 patients and tumor overgrowth occurred in 8 patients. The mean dysphagia score of 2.8 improved to a mean score of 1.0 after stenting (*P* < 0.001) [19]. In another case series of 66 patients with unresectable esophageal cancer, the SEPS Polyflex was used; in all patients in this study, the insertion of SEPSs led to an improvement in dysphagia. Additionally, the rate of tumor overgrowth and stent migration were low in this cohort [20]. 

All SEMS are delivered in a compressed form, which allows the delivery system to have a relatively small diameter compared with the final stent diameter after deployment. Many of the stents are preloaded onto the delivery system, though some require loading immediately prior to the procedure. The delivery systems vary in size from 6 to 14 mm. All delivery system have radio-opaque markers indicating the limits of the compressed stent as well as the expected stent length when fully expanded. Below is an overview of some of the most common SEMS and SEPS available.

- Ultraflex (Boston Scientific, Boston, Mass, USA) is a construction mesh SEMS knitted from a single strand of nitinol wire; it is available in two forms: uncovered and the covered, where the body of the stent is covered by sheath of polyurethane. The stent is kept compressed along a supple plastic guide by means of a coiled thread around the stent. Once this thread is pulled, the stent self-expands to its final diameter. It may be placed without fluoroscopy by using a ruler on the shaft of the delivery system to measure visual markers. It is an extremely flexible SEMS but has the lowest expansile force of all available metal prostheses; as a result, the stent may need to be dilated with a balloon to achieve adequate expansion.

(ii) Flamingo Wallstent (Boston Scientific, Boston, Mass, USA) has a conical or funnel-shaped design, constructed from a braided stainless steel alloy. This conical shape provides greater radial expansion proximally, in order to reduce migration across the esophagogastric junction. This is an older device and is no longer available in the USA.

(iii) Wallflex (Boston Scientific, Boston, Mass, USA) is a newer generation of SEMSs. It is available as fully and partially covered stent. Unlike other stents, the fully covered the Wallflex may be reconstrained up to 75% of deployment and up to two times during initial stent placement procedure. It also incorporates a purse string Teflon coated polyester suture at the proximal end which facilitates stent repositioning or removal. This stent also has “progressive step flared ends” that theoretically reduces migration by anchoring the stent within the esophageal lumen.

(iv) Evolution (Cook, Bloomington, Ind, USA) is available as a partially or fully covered SEMS. The stent is encased with silicone on its exterior and interior surfaces to prevent tumor ingrowth. Additionally, the fully covered version incorporates a dual purse string “lasso loop” on the proximal and distal ends of the stent to aid in repositioning, if needed. A unique feature of Evolution delivery system is that it enables a controlled release and recapturability feature with a “point of no return” indicator. With each squeeze of the stent system's trigger-based introducer, a proportional length of the stent is deployed or recaptured.

(v) Alimaxx-E (Merit Medical Systems, UT) is a laser cut nitinol stent, fully coated with polyurethane on the exterior and silicone lining. It contains “antimigration struts” that project along the length of the stent in an effort to prevent migration.

(vi) Niti-S (Taewoond Medical, Korea) has a double-layer configuration consisting of an inner polyurethane layer to prevent tumor overgrowth and an outer uncovered nitinol wire tube to allow the mesh of the stent to embed itself in the esophageal wall.
(vii) Polyflex (Boston Scientific, Boston, Mass, USA) consist of polyester netting completely covered in a silicone membrane. The device must be loaded onto the delivery system prior to deployment.

## 5. Technique of Insertion

In general, image guidance with plain X-ray fluoroscopy is sufficient for successful stent placement, while a hybrid approach with endoscopy may be used in technically challenging cases. In our institution, we prefer the hybrid approach. The patient is placed supine and either moderate conscious sedation or general anesthesia is induced. Flexible esophagoscopy is performed to characterize the lesion. Then, using fluoroscopic guidance, the proximal and distal extent of the tumor is marked on the skin using radiopaque metal markers. If the endoscope cannot be passed through the tumor, dilation is performed. A guidewire is placed through the tumor, and the delivery system is inserted over the wire. The stent is deployed across the tumor length previously delineated by the radiopaque markers. Balloon dilation of the stent may be performed if the stent has a low expansile force and has not expanded sufficiently. 

## 6. Postprocedure Care

After placement of any esophageal stent and before reinstitution of oral feeding, an X-ray of the chest and a contrast-enhanced esophagography with dilute barium are performed to rule out perforation and to ensure the correct position of the prosthesis. Nutritional instructions are given to the patients to avoid food impaction within the stents. Usually, patients are allowed oral feeding 12 hours post-procedure in order to allow adequate time to detect any procedure-related complications. Diet is usually started as soft or liquid; diets high in fiber and large meals are avoided. Drinking with meals is recommended to wash away any food remnants. Patients with esophageal stents should not lie down flat; they are advised to eat in an upright position and to sleep at a 30-degree angle. A proton pump inhibitor is recommended for all patients who develop reflux after stent deployment and in those where the stent is placed across the GE junction [6]. 

## 7. Clinical Outcomes

The optimum management of malignant dysphagia has evolved over the past two decades, with SEMS playing a greater role in the palliation of advanced esophageal cancer. Since the early 1990s, metal stents have replaced rigid plastic stents as they have been found to be more cost effective and safer, with fewer associated complications [12, 13, 21]. In a randomized controlled, prospective trial by Knyrim and colleagues, 39 patients with esophageal carcinoma and 3 with malignant extrinsic obstruction were randomized to either a 16 mm diameter plastic prosthesis (Wilson-Cook, Winston-Salem, NC, USA) or an expandable metal-mesh stent (Wallstent, Schneider AG, Switzerland) made of stainless steel. In this study, the complication rates were significantly less frequent with the metal stents than with the plastic prostheses (0 versus 9; *P* < 0.001). There were similar degrees in improvement in dysphagia and Karnofsky score between treatment arms. The most common causes of recurrent dysphagia were migration of the plastic prostheses (*n* = 5 patients), or an ingrowth or overgrowth of the metal stents by tumor (*n* = 5 patients). The authors concluded that despite having a higher initial cost, metal stents were ultimately more cost effective because of the absence of fatal complications and the decrease in the hospital stay associated with their use [13]. In another randomized, controlled study, 31 patients with inoperable malignant esophageal stenosis causing dysphagia were randomized to receive either a modified Gianturco SEMS or a plastic Atkinson tube; again,there were similar complication rates observed between the two groups. Nevertheless, compared to patients who received Atkinson plastic tubes, patients with Gianturco SEMS had better palliation of dysphagia (median dysphagia score 1 versus 2, *P* = 0.04), maintained their weight longer, had shorter hospital stays, and had a longer survival rate [22]. A more recent retrospective review of 153 patients (45 with plastic prostheses and 108 with SEMS) also showed an improvement in dysphagia, survival, with comparable recurrent dysphagia rates between the two groups. However, significantly more major complications were seen in the plastic prosthesis group compared with the SEMS group [23]. In summary, SEMS have shown to be superior to rigid plastic prostheses in the palliative management of unresectable obstructive esophageal cancers [7]. 

The ease of insertion and high technical success rate associated with SEMS account for their widespread use [24]. They may provide instant dysphagia relief in up to 96% of patients while improving dysphagia scores by 1-2 grades. Despite the various types of stents available there is no apparent superiority among different stents with regards of dysphagia relief [21, 25–30]. In a prospective randomized controlled trial, Sabharwal and colleagues compared the rate of early and late complications (perforation, migration, severe gastroesophageal reflux, hematemesis, and reobstruction due to tumor overgrowth) in 53 patients diagnosed with inoperable lower third esophageal carcinoma randomized to receive either a flamingo covered wallstent (Boston Scientific Inc., Watertown, Mass, USA) or an Ultraflex covered stent (Boston Scientific Inc.) for palliation. In both stent groups, there was a significant improvement in dysphagia (*P* < 0.05) but no significant difference was seen between the two groups (*P* > 0.1). The frequency of complications among the groups was similar [31]. In another prospective randomized study, Siersema compared the placement of an Ultraflex stent, a Flamingo Wallstent or a Gianturco-Z stent in a group of 100 patients with esophagogastric carcinoma. All 3 covered metal stents offered the same degree of palliation (*P* < 0.001) with no difference in the degree of improvement among the groups (*P* = 0.014). Placement of Gianturco-Z stents, although not statistically significant (*P* = 0.23), was associated with more complications (36%) compared with Ultraflex stents (33%) and Flamingo Wallstents (24%) [32]. 

Palliative stenting is associated with a reintervention rate of approximately 25%–35%, requiring close followup of these patients in order to maintain effective palliation. The median and mean survival after stent placement ranges from approximately 78–83 days and 120–126 days, respectively, regardless of the type of stent used. Survival has not been associated with the etiology of the malignancy or the type of stent used; rather, it is related to the progression of disease [9, 12]. 

While typically used for palliation in patients with unresectable or metastatic disease, esophageal stenting has also been used in patients receiving neoadjuvant therapy for patients awaiting definitive surgical treatment. Compared to feeding tubes, removable self-expanding silicone stents allows for maintenance of preoperative enteral nutrition while avoiding the need for operatively placed j-tubes. In some small series, its use in the neoadjuvant setting has been shown to be safe and effective without an adverse effect on intraoperative dissection, perioperative complications, or delay in resection after neoadjuvant therapy [33–35]. 

## 8. Comparing Self-Expandable Metal (SEMSs) and Self-Expandable Plastic (SEPSs) Stents

One of the drawbacks of the currently generation of stents is the phenomenon of recurrent dysphagia due to stent migration and tissue growth. Tumor ingrowth, initially noted in first generation of uncovered SEMS, led to the development of other options including new self-expandable plastic stents (SEPS). A recent prospective, randomized controlled trial compared the new SEPS (Polyflex) with SEMS (Ultraflex) in a 100 patients with unresectable esophageal carcinoma; stent placement was successful in 98% and 100%, respectively. In multivariate analysis, there was a significantly higher complication rate with Polyflex than with Ultraflex stents (odds ratio 2.3, 95% CI 1.2–4.4). No difference was seen in palliation of dysphagia between the two stents. The median survival was 134 days with Polyflex and 122 days with Ultraflex stents (*P* = NS). In another study, 125 patients with dysphagia from inoperable carcinoma of the esophagus or gastric cardia were randomized to placement of an Ultraflex (*N* = 42), Polyflex stent (*N* = 41), or Niti-S stent (*N* = 42). Improvement of dysphagia was comparable between all three stent groups. However, recurrent dysphagia, caused by tissue ingrowth, migration, or food obstruction, was significantly different between patients with an Ultraflex stent and patients with a Polyflex stent or Niti-S stent (52% versus 37% versus 31%, *P* = 0.03). Stent migration occurred more frequently with Polyflex stents which were also associated with more technical difficulties during actual stent placement. 

The rate of tissue overgrowth was more higher with Ultraflex stents, and to a lesser degree, Niti-S stents, but not statistically significant [36]. No difference in survival was found between cohorts. On the basis of the technical difficulties and high stent migration rates, the investigators concluded that SEPS was the least preferable stent in this patient group. The majority of recent studies suggest that despite the comparable efficacy in the treatment of dysphagia between SEMSs and SEPSs, SEMSs are associated with significantly fewer complications than SEPSs for the palliation of malignant dysphagia. 

## 9. Comparing SEMSs with Other Modalities

Despite the advances in therapeutic modalities and techniques, the optimal method of palliation for unresectable esophageal cancer patients remains uncertain and is not standardized. Compared to plastic tube insertion, SEMS provide better palliation of dysphagia, reduced recurrent dysphagia, decreased initial hospital stay, and procedure-related morbidity and mortality [5, 6, 12, 13]. Thermal and chemical ablative modalities such as photodynamic therapy (PDT) and laser provide a level of palliation comparable to that achieved with SEMSs. However, the increased rate of reinterventions associated with their use makes PDT a less desirable option [37]. Brachytherapy has been found to palliate dysphagia effectively in patients with advanced esophageal cancer, providing a sustained improvement in dysphagia symptoms and quality of life over time. Brachytherapy is also associated with a low incidence of complications, making it a very suitable alternative to SEMS insertion in the palliation of patients with advanced esophageal and gastroesophageal junction cancers. However, this difference gradually diminishes after 12 months and the optimal dosage and fractional schedule is still uncertain [38–40].

## 10. Complications

Insertion of SEMS is not without its share of complications. While early complications are relatively uncommon, delayed complications can occur in up to 53%–65% of patients, with a reintervention rate as high as 50% [41–43]. Approximately 0.5%–2% of patients die as direct result of expandable stent placement. Prior radiotherapy or chemotherapy contributes to a significantly higher rate of life-threatening complications and mortality rate after stent insertion [44, 45]. 

Some of the most common early procedure-related complications include chest pain, bleeding, perforation, and aspiration. Prolonged chest pain is reported in 12%–14% of cases after SEMS insertion and is more prominent when stents are placed in the upper cervical esophagus, and with stents having either a high radial force, such as Wallstent, or a flared design such as the Flamingo stent. A small amount of bleeding 24 to 48 hours after SEMSs insertion is relatively common and is usually due to direct trauma during the procedure. Lung aspiration secondary to chronic gastroesophageal reflux can occur during stent placement and as a result of stent positioning across one of the esophageal sphincters. These problems can be addressed with the newer regeneration of stents, specifically those with an antireflux valve, coupled with strict medical management of GERD with aggressive acid suppression and antireflux precautions. The risk of perforation or stent erosion into adjacent structures is rare but has been described. It occurs more frequently in patients whose tumors were previously treated with chemotherapy, radiation, or laser therapy. When recognized early it can be treated with a covered stent and administration of antibiotics [22, 41, 43, 45–48]. 

Delayed complications include tumor ingrowth or overgrowth, stent migration, recurrent dysphagia, intractable reflux, tracheoesophageal fistula, bleeding, and perforation. Stent obstruction with recurrent dysphagia may be due to tumor progression, reactive hyperplasia or food impaction [45]. Tumor ingrowth through the mesh is most commonly seen in uncovered stents and should not occur in covered SEMS. Overgrowth of the stent by tumor may occur in 4%–18% of cases [43]. It can occur at either end of a covered or an uncovered stent, underlying the importance of choosing the appropriate stent length (with a minimum of 2 cm beyond the proximal and distal margins of the tumor). In such cases, tumor overgrowth may be treated with ablative therapies such as laser and photodynamic therapy or with a second stent deployment. Covered stents have a reportedly higher incidence of stent migration with series reporting as high as 23% compared to uncovered stents, which have a rate of approximately 8.7% [22, 42, 46]. It is usually more common when the stent is positioned across the gastroesophageal junction. Other factors contributing to stent migration are stent malposition, shrinkage of the tumor from chemotherapy or radiation therapy, or overdilation of the lesion before stent placement [45]. Stent removal either surgically or endoscopically after complete migration is only indicated for symptomatic patients [49]. Partial stent migration is treated with coaxial insertion of another overlapping stent. A few cases of fistula formation and tracheal compression have been reported, especially in case of stent placement in the upper and mid-third of the esophagus [50–52]. Bleeding can also occur due to stent erosion into vessels within the esophageal wall. Perforation may occur and is thought to result from pressure necrosis caused by the continuing radial force of the stent on the tumor or from the stent struts penetrating through the esophageal wall. 

## 11. Future Technology

There are a number of future trends on the horizon with regards to stent technology. Removable SEPSs have been introduced for use in benign and malignant strictures, as well as for postesophagectomy complications. Biodegradable stents made of polylactic polymers have recently been utilized, and though experience with this is limited, they have shown some efficacy in the management of refractory benign esophageal strictures after endoscopic submucosal dissection [53–55]. More recently, drug-eluting esophageal stents have introduced in treatment of refractory benign and malignant strictures, as they provide mechanical support as well as release of drugs to prevent restenosis by inhibiting tissue hyperplasia [56, 57]. 

## 12. Conclusion

Palliation of esophageal malignant dysphagia can be challenging and often requires a multidisciplinary approach for optimal success. Esophageal stenting has been shown to be a safe and effective tool for refractory dysphagia. Recent advances in stent technology have reduced peri- and postprocedural complications, resulting in improved quality of life for these patients. Because of this, esophageal stents have become the therapy of choice for palliation of dysphagia with further investigation aimed at providing continued and prolonged relief of symptoms. 

## Figures and Tables

**Table 1:** Indications and contraindications for stent use in esophageal obstruction due to malignancy.

Indications	

Unresectable malignant esophageal obstruction	
Extrinsic esophageal compression by primary or secondary mediastinal tumors	
Actual or impending fistula	
Malignant gastroesophageal anastomotic leaks	
Tumor recurrence after surgery or chemoradiotherapy	
Contraindication to chemoradiotherapy	

Contraindications	

Curable disease by multimodality treatment (relative)	
Tumor or stricture within 2 cm of proximal esophageal sphincter	
Uncorrectable coagulopathy	
Potential for significant airway compression	
Recent high-dose chemoradiotherapy (within 3–6 weeks)	
Terminal ill patient with limited life expectancy	

Sources: [5, 6].

**Table 2:** Esophageal stents currently on the market in the USA, Europe, and Asia.

Stent	Manufacturer	Material	Covering	Length (cm)	Diameter shaft/flare (mm)		Antireflux valve	FDA approved
Alimaxx-ES	Alveolus	Nitinol	FC	7/10/2012	18,22		No	Yes

Choostent	MI Tech	Nitinol	FC	5 ~ 20	18,20,22		Yes (valve variant)	Yes

Esophageal Z	Cook	Stainless steel	PC/FC	8/10/12/14	18/25		Yes (Dua variant)	Yes

Evolution	Cook	Nitinol	PC	8/10/12.5/15	18/23 (FC)		No	Yes
				(PC) 8/10/12 (FC)	20/25 (PC,FC)			

FerX-Ella	Ella-CS	Stainless steel	FC	9/10.5/12/13.5 15/16.5/18/ 19.5 /21	20/36		Yes/No	No

Flamingo Wallstent	Boston Scientific	Stainless steel	PC	12/14	20/30		No	No

Gianturco Z	Cook	Stainless steel	PC	8/10/12/14	18/25		Yes	No
							No; shaft bars	No

Niti-S	Taewoong Medical	Nitinol	PC/FC	3/5/7/9/12	16/24		Yes (PTFE variant)	Yes
					18/26			
					20/28			

Polyflex	Boston Scientific	Polyester	FC	9/12/2015	16/20		No	Yes
					18/23			
					21/28			

Ultraflex NG	Boston Scientific	Nitinol	NC/PC	7/10/12/15	18,23		No	Yes

Wallflex	Boston Scientific	Nitinol	PC/FC	10/12/15	PC 18/23	FC 18/25	No	Yes
					23/28	23/28		

NC, not covered; PC, partially covered; FC, fully covered; FDA, Food and Drug Administration.
